# Prevalence of personal weight control attempts in adults: a systematic review and meta‐analysis

**DOI:** 10.1111/obr.12466

**Published:** 2016-09-21

**Authors:** I. Santos, F. F. Sniehotta, M. M. Marques, E. V. Carraça, P. J. Teixeira

**Affiliations:** ^1^ Interdisciplinary Centre for the Study of Human Performance (CIPER), Faculty of Human KineticsUniversity of LisbonLisbonPortugal; ^2^ Institute of Health and SocietyMedical Faculty of Medical Sciences, Newcastle UniversityNewcastle upon TyneUK; ^3^ Fuse, the UK CRC Centre of Excellence for Translational Research in Public HealthNewcastle upon TyneUK

**Keywords:** Maintenance, motives, strategies, weight loss

## Abstract

The purpose of this systematic review and meta‐analysis was to estimate the prevalence of personal weight control attempts (weight loss and/or maintenance) worldwide and to identify correlates, personal strategies used and the underlying motives. We included epidemiological/observational studies of adults (≥18 years) reporting prevalence of weight control attempts in the past‐year. Seventy‐two studies (*n* = 1,184,942) met eligibility criteria. Results from high quality studies showed that 42% of adults from general populations and 44% of adults from ethnic‐minority populations reported trying to *lose* weight, and 23% of adults from general populations reported trying to *maintain* weight annually. In general population studies, higher prevalence of weight *loss* attempts was observed in the decade of 2000–2009 (48.2%), in Europe/Central Asia (61.3%) and in overweight/obese individuals and in women (*p* < 0.01). Of the 37 strategies (grouped in 10 domains of the Oxford Food and Activity Behaviours Taxonomy) and 12 motives reported for trying to control weight, exercising and dieting (within the energy compensation and restraint domains, respectively) and wellbeing and long‐term health were the most prevalent. To our knowledge, this is the first systematic review to investigate weight control attempts worldwide. Key strategies and motives were identified which have implications for future public health initiatives on weight control.

## Introduction

The causes of obesity are complex and multifaceted. Obesity control interventions usually focus on a combination of physical and dietary aspects of social, economic and cultural environments together with individual approaches 1, 2. Personal weight control efforts (i.e. intentionally trying to lose or maintain weight) are of particular relevance for public health as they reflect an active investment of the population and provide an opportunity to guide individuals to evidence‐based weight control approaches.

In other areas of public health (e.g. smoking cessation), a focus on personal behaviour change attempts (e.g. quit smoking) has been key to understanding and enhancing effects of public health strategies 3. The relationship between personal weight loss attempts and obesity is complex, and well‐informed attempts to lose weight (e.g. those utilizing evidence‐based weight loss strategies) may result in better weight loss and maintenance 1, 4. However, there is also consistent evidence that amongst obese adults the number of weight loss attempts is a negative predictor of success in weight loss interventions 5. Recurrent weight control efforts may negatively impact on self‐concept, body image, pessimistic attributions and feelings of helplessness, all of which could predispose individuals to failure 6, 7. Therefore, it is important to understand *how many* weight control attempts are made, by *whom*, *how* and *why*, in order to provide a clearer knowledge base about what people seeking weight control are currently doing (and why) and inform public health policies and interventions regarding changes that need to occur in weight loss/maintenance attempts to improve population outcomes.

This systematic review and meta‐analysis aimed to (i) synthesize the available epidemiological data on the prevalence of weight control (weight loss and weight maintenance) attempts among adults worldwide; (ii) provide a comprehensive description of the personal strategies used and (iii) describe the motives behind those attempts. To our knowledge, this is the first study providing such a perspective.

## Methods

This systematic review and meta‐analysis is reported in accordance with *The Joanna Briggs Institute Reviewers*' *Manual 2014* for systematic reviews of prevalence and incidence data 8. Methodological aspects of this review were specified in advance and documented in a protocol (PROSPERO registration number: CRD42014010572).

### Eligibility criteria

Studies were selected for this review if they were population‐based epidemiological/observational studies that included samples of adults (≥18 years old). To be eligible, studies should also include a question on the prevalence of weight control (loss and/or maintenance 1Maintenance does not necessarily imply previous weight loss.) attempts within a 12‐month period preceding the survey (e.g. ‘Are you currently trying to lose weight?’, ‘Have you tried to lose weight in the past 6 months?’, ‘Have you tried to lose weight in the last year?’, ‘Are you now trying to maintain your weight, that is, to keep from gaining weight?’, ‘Have you tried to keep from gaining weight during the previous 12 months?’). Past year prevalence was chosen instead of ever prevalence because it has a greater potential to reflect changing patterns over time and capture differences (e.g. between geographical regions). Studies of pregnant women (or women within 1‐year postpartum), athletes and populations with specific health conditions, disabilities or mental disorders were excluded.

### Search strategy and study selection

A comprehensive search of peer‐reviewed articles was conducted in three electronic databases: PubMed, PsycInfo and Web of Science (all articles published until December 2015). Searches included various combinations of the following terms: weight control, weight loss, weight maintenance, diet, attempts, prevalence, strategies, practices, determinants and motives (Full search strategy is available from the authors upon request). The search was limited to studies with participants aged 18 years and older. There were no restrictions regarding the language of publication. Additionally, manual cross‐referencing of retrieved articles and hand‐searches of key scientific journals (e.g. *International Journal of Public Health*, *American Journal of Preventive Medicine*) were performed.

Potentially eligible studies were independently identified by two authors (IS, EVC), based on titles, abstracts and references. Duplicate entries were removed. Relevant articles were then retrieved for a full‐text review. The same two researchers independently reviewed the full‐text of potential studies and discrepancies were resolved by consensus. Endnote® X7 for Mac® OS X® was used to manage the references.

### Methodological quality

The methodological quality of included studies was assessed using a standardized form based on a short version of *The Joanna Briggs Institute critical appraisal checklist for studies reporting prevalence data* 8, consisting of a five‐category tool addressing critical issues of internal and external validity of prevalence data, including (i) representativeness of the sample; (ii) appropriate recruitment of study participants; (iii) adequacy of sample size; (iv) non‐response and refusals and (v) use of a standard criteria for the measurement of the condition. For each study, each category of the checklist was classified as *Yes*, *No*, *Unclear* or *Not applicable*. *No* corresponds to a limitation in the respective methodological category. Two of three researches (IS, MM and EVC) independently assessed the methodological quality of each study and discussed the results of their critical appraisals. Disagreements were resolved by consensus.

### Data extraction

*The Joanna Briggs Institute data extraction form for prevalence and incidence studies* 8 was used to extract relevant information. Data extraction included information about (i) study details (authors, year, publication journal); (ii) study methods (design, mode of data collection, year of survey, geographical region, setting); (iii) subject characteristics (sample size, age, gender, percentage of overweight/obesity, response rate) and (iv) outcomes of interest (prevalence of weight loss and maintenance attempts, strategies used and motives reported by those trying to control their weight).

### Data synthesis and statistical analyses

We conducted separate meta‐analyses for the prevalence of weight loss and weight maintenance attempts in (i) general populations and (ii) ethnic‐minority populations.

Analyses were conducted using the Comprehensive Meta‐Analysis Software version 2.2 9. Meta‐analyses were conducted using random‐effects models, in which the summary effect is an estimate of the mean of a distribution of effect sizes 10. Pooled effects were the prevalence estimates of weight loss and maintenance attempts (represented as event rate plus confidence intervals). To evaluate the amount of variation in the effects of included studies, we inspected for heterogeneity using: (i) the Cochran's *Q* statistic 11, for which a significant *p*‐value (<0.05) demonstrates that studies do not share a common effect size (i.e. there is heterogeneity in the effect sizes between studies); and (ii) *I* ^2^ statistic 12 that assesses the proportion of observed dispersion that is because of real differences in the actual effect sizes (rather than sampling error). The *I* ^2^ ranges from 0 to 100%, where a value of 0% indicates no observed heterogeneity and values of 25%, 50% and 75% reflect low, moderate and high heterogeneity, respectively.

Subgroup analyses were conducted to examine whether prevalence estimates varied according to the decade of the survey (1970–1979, 1980–1989, 1990–1999, 2000–2009 and 2010–2015) and the geographic region where the survey took place (coded according with the World Bank Atlas as Africa, East Asia and Pacific, Europe and Central Asia, Latin America and the Caribbean, Middle East and North Africa, North America and South Asia). These subgroup analyses were conducted using mixed‐effect models (i.e. random‐effects model is conducted within subgroups and a fixed effect model was used across subgroups) 10. Between‐groups *Q* statistic and corresponding *p*‐values were used to compare the mean effect across subgroups. Further, meta‐regressions using mixed‐effects models were conducted to analyse the moderation effect of the following continuous variables: (i) percentage of overweight and obese individuals in the sample; (ii) percentage of women in the sample and (iii) mean age of the sample. Meta‐regressions were conducted when there were at least 10 studies/analyses and were analysed based on the *Z*‐value and associated *p*‐value of the slope 10. Because of the limited number of studies reporting the prevalence of weight maintenance attempts, we only conducted moderator analyses for the prevalence of weight loss attempts.

Some studies did not provide separated prevalence rates of weight loss and maintenance attempts and did not include sufficient data (e.g. mean age) for subgroup analyses and meta‐regression. Therefore, the number of studies included in moderation analyses varies.

Personal weight control strategies and motives reported by those trying to control their weight in the past year were qualitatively synthetized and presented in tabular form. Personal weight control strategies were independently classified within the domains of the Oxford Food and Activity Behaviours (OxFAB) Taxonomy 13 by two of three researchers (IS, MM and EVC) and discrepancies were resolved by consensus. This taxonomy was chosen because it is a comprehensive tool to systematically describe the cognitive and behavioural strategies used by individuals for weight management 13. Only the domains where at least one strategy fell on were shown. Two additional domains were included – dietary choices and extreme strategies – as some of the reported strategies did not fit within any existing domain. Likewise, some strategies seemed to fit in more than one domain; nevertheless, we have selected the one that appeared more appropriate. Weight control motives were independently extracted by two of three researchers (IS, MM and EVC).

### Sensitivity analyses

Sensitivity analyses were carried out to explore if overall results were affected by methodological quality. Primary analyses were repeated excluding studies presenting methodological limitations in either and in all (cumulative) categories of the *The Joanna Briggs Institute critical appraisal checklist for studies reporting prevalence data* 8. Moderation analyses were also repeated excluding all studies presenting methodological limitations.

Publication bias was examined by (i) visual inspection of funnel plot and asymmetry and (ii) Egger's test 14 to confirm the visual impression.

## Results

**Figure 1:**
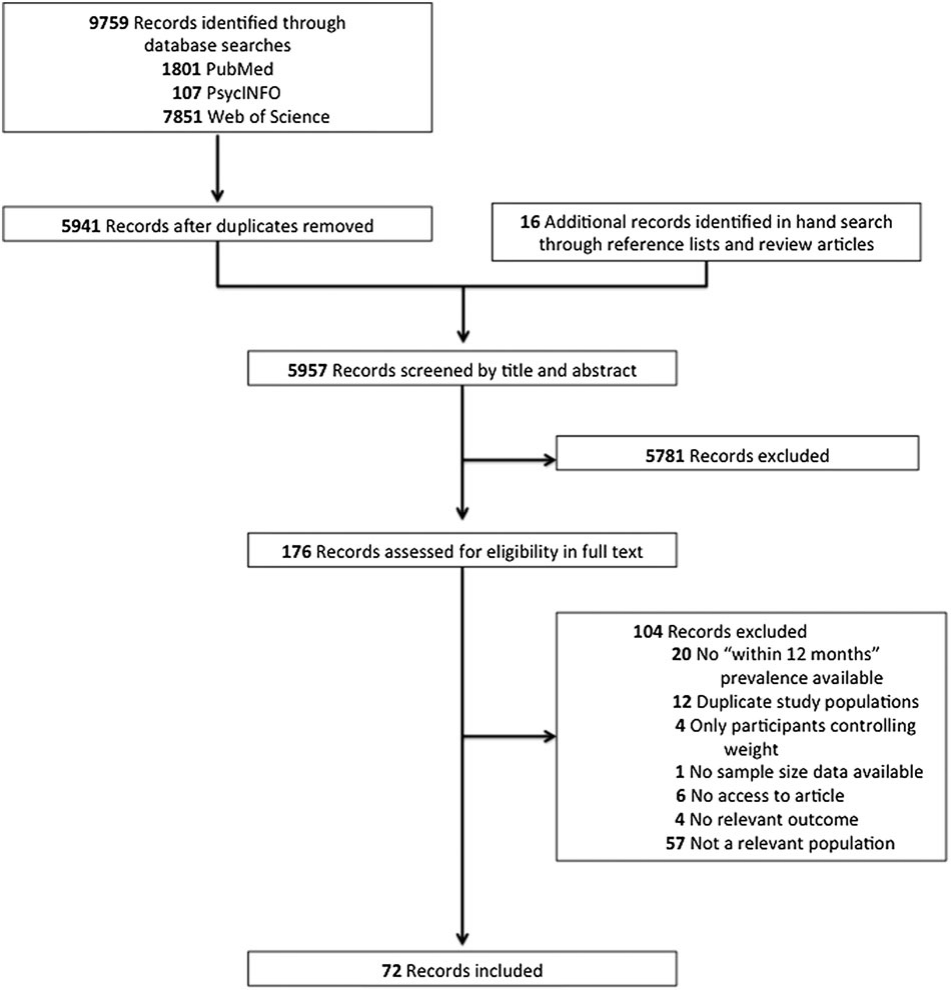
Flow diagram of studies.

The literature search yielded a total of 9,759 records. Sixteen articles identified through manual searches and cross‐referencing were added, leading to a total of 9,775 potential articles (Fig. 1). After duplicate removal (*n* = 3,818), 5,957 articles were assessed for eligibility. Of these, 5,781 were excluded based on title/abstract screening, leaving 176 eligible for full‐text screening. Seventy‐two articles with a total sample size of 1,184,942 met eligible criteria and were included.

### Study and sample characteristics

Characteristics of included studies are summarized in Table 1. Sixty studies were conducted within general populations and 12 within ethnic‐minority populations. Most studies had a cross‐sectional design (*k* = 67), and the remaining five studies were prospective cohorts. Surveys were conducted between 1975 and 2013 across 40 countries within five continents, and data was collected via in‐person/self‐administered (*k* = 41), telephone (*k* = 17), mail (*k* = 11) and online (*k* = 3) surveys. Sixty‐two studies included mixed‐gender samples, nine included only women and one study was conducted only with men. Eleven studies targeted overweight and obese individuals only. Sample sizes in the studies ranged from 123 to 170,971 participants and response rates from 24% to 97.7%.

**Table 1:** Studies on the prevalence of weight control attempts sorted by country

								Prevalence of weight control attempts (%)				
Reference	Study design	Data collection	Year of survey	Population	Sample size/% women	Age (range; mean ± SD (years))	%Overweight^1^/Obesity^2^	Trying to lose	Trying to maintain^4^	Total	Response rate (%)	Methodological limitations (categories)
(**a**)												
15	PCS	MS	1993	Australia reg.	*n* = 1,342/50.4	≥18; —	38.2/15.9	20.4	22.2	42.6	67.8	—
16	CSS	MS	1997	Australia reg.	*n* = 900/57.9	≥18; 44.7 ± 16.5	30.0/12.7	22.9*	26.3*	49.2*	41.6	3
17	CSS	IS	2004	Australia spec.^a^	*n* = 1,973/58.0	≥18; —	33.4/23.2	37.0	—	—	—	4
18	PCS	MS	2007–2008	Australia spec.^b^	*n* = 1,634/100	18–46; 36.5 ± 7.6	—	39.1	29.9	69.0	45.0	3, 4
19	CSS	OS	2010–2011	Australia spec.^a^	*n* = 1,335/60.9	≥18; —	35.4/22.8	50.0	—	—	85.0	1, 3
20	CSS	IS/MS	—	Australia spec.^c^	*n* = 994/54.0	≥18, —	28.4/8.4	46.9	—	—	65.4	4
21	CSS	IS	2013	Bangladesh spec.^d^	*n* = 649/—	16–30; 20.8 ± 2.8	34.2^3^	32.1***	—	—	—	1, 3
21	CSS	IS	2013	Barbados spec.^d^	*n* = 577/—	16–30; 20.8 ± 2.8	40.0^3^	11.4***	—	—	—	1, 3
22	CSS	MS	2001	Belgium nat.	*n* = 2,591/62.6	18–74; —	23.4/9.8	—	—	46.4	—	—
23	CSS	IS	2010	Brazil reg.	*n* = 2,732/57.9	≥20; 46.1 ± 17.0	36.3/26.0	26.6*	—	—	89.3	—
24	CSS	IS	1986–1992	Canada nat.	*n* = 17,564/50.9	18–74; —	34.3/15.4	32.6*	—	—	66.5	—
25	CSS	OS	2008	Canada spec.^d^	*n* = 3,069/74.8	—; 27.9 ± 10.2	25.6^3^	33.3	—	—	—	1, 2, 3, 4
21	CSS	IS	2013	Colombia spec.^d^	*n* = 810/—	16–30; 20.8 ± 2.8	25.5^3^	18.8***	—	—	—	1, 3
21	CSS	IS	2013	Egypt spec.^d^	*n* = 696/—	16–30; 20.8 ± 2.8	41.6^3^	20.5***	—	—	—	1, 3
26	CSS	IS	1997–1998/2002	England nat.	*n* = 9,098/48.1	25–60; —	100^3^	66.5*	—	—	—	—
27	PCS	MS	1975	Finland nat.	*n* = 7,729/54.3	18–54; —	—	18.6*	—	—	89.0	4
28	CSS	IS	1990–1991	France spec.^d^	*n* = 656/55.6	18–30; 21.4 ± 2.5	1.8/0.3	23.8	—	—	82.0	1, 2, 3, 4
29	CSS	IS	1999	Great Britain nat.	*n* = 1,894/50.5	—; 45.8 ± 18.2	32.3/10.8	28.5	36.4	64.9	70.0	4
30	CSS	IS	2012	Great Britain nat.	*n* = 810/46.7	16–90; 51.3 ± 17.9	65.2/34.8	45.0	—	—	—	—
21	CSS	IS	2013	India spec.^d^	*n* = 800/—	16–30; 20.8 ± 2.8	36.8^3^	16.4***	—	—	—	1, 3
22	CSS	MS	2001	Italy nat.	*n* = 1,062/42.4	18–74; —	29.2/6.8	—	—	46.5	—	—
21	CSS	IS	2013	Ivory Coast spec.^d^	*n* = 777/—	16–30; 20.8 ± 2.8	11.7^3^	18.1***	—	—	—	1, 3
21	CSS	IS	2013	Jamaica spec.^d^	*n* = 675/—	16–30; 20.8 ± 2.8	27.9^3^	14.3***	—	—	—	1, 3
31	CSS	IS	1998	Japan spec.^e^	*n* = 146/0	30–65; 47.5 ± 9.3	30.8^3^	32.2	—	—	80.1	1, 2, 3, 4
21	CSS	IS	2013	Kyrgyzstan spec.^d^	*n* = 814/—	16–30; 20.8 ± 2.8	9.2^3^	23.1***	—	—	—	1, 3
21	CSS	IS	2013	Laos spec.^d^	*n* = 759/—	16–30; 20.8 ± 2.8	20.6^3^	9.5***	—	—	—	1, 3
32	CSS	IS	2001	Lebanon spec.^d^	*n* = 2,013/60.0	—; 21.0 ± 2.4	18.0^3^	30.0	—	—	90.0	1, 3
21	CSS	IS	2013	Madagascar spec.^d^	*n* = 780/—	16–30; 20.8 ± 2.8	5.1^3^	21.6***	—	—	—	1, 3
33	CSS	IS	2009	Malaysia spec.^e^	*n* = 233/55.4	18–60; 32.5 ± 10.5	29.6/20.6	73.8	—	—	70.6	4
34	CSS	IS	—	Malaysia spec.^f^	*n* = 1,032/62.8	≥18; —	—	24.5	—	—	—	1, 2, 3, 4
21	CSS	IS	2013	Mauritius spec.^d^	*n* = 461/—	16–30; 20.8 ± 2.8	13.0^3^	16.4***	—	—	—	1, 3
35	CSS	IS	2004	Mexico spec.^d^	*n* = 2,651/62.0	17–45; 20.2 ± 2.6	28.9^3^	38.8	—	—	—	1, 3, 4
21	CSS	IS	2013	Namibia spec.^d^	*n* = 466/—	16–30; 20.8 ± 2.8	19.3^3^	16.4***	—	—	—	1, 3
36	CSS	MS	2009	New Zealand nat.	*n* = 1,601/100	40–50; 45.5 ± 3.2	29.4/20.7	39.4	42.1	81.5	65.8	—
21	CSS	IS	2013	Nigeria spec.^d^	*n* = 800/—	16–30; 20.8 ± 2.8	13.3^3^	12.7***	—	—	—	1, 3
37	PCS	MS	1997	Norway nat.	*n* = 10,025/100	45–59; —	31.0/8.0	51.6	—	—	51.2	2
21	CSS	IS	2013	Pakistan spec.^d^	*n* = 761/—	16–30; 20.8 ± 2.8	15.0^3^	27.7	—	—	—	1, 3
21	CSS	IS	2013	Philippines spec.^d^	*n* = 769/—	16–30; 20.8 ± 2.8	22.5^3^	24.5***	—	—	—	1, 3
22	CSS	MS	2001	Portugal nat.	*n* = 1,313/55.4	18–74; —	32.9/9.3	—	—	37.0	—	—
38	CSS	TS	2012	Portugal nat.	*n* = 1,098/48.5	18–65; 40.1 ± 13.3	34.0/10.8	24.3	19.4	43.7	57.9	—
21	CSS	IS	2013	Russia spec.^d^	*n* = 785/—	16–30; 20.8 ± 2.8	17.0^3^	22.0***	—	—	—	1, 3
21	CSS	IS	2013	Singapore spec.^d^	*n* = 678/—	16–30; 20.8 ± 2.8	22.1^3^	26.8***	—	—	—	1, 3
21	CSS	IS	2013	South Africa spec.^d^	*n* = 749/—	16–30; 20.8 ± 2.8	30.7^3^	15.3***	—	—	—	1, 3
22	CSS	MS	2001	Spain nat.	*n* = 3,543/52.6	18–74; —	33.5/11.6	—	—	43.3	—	—
39	CSS	IS	2012	Thailand spec.^d^	*n* = 860/72.7	18–25; 20.1 ± 1.3	7.8/13.0	42.5	—	—	97.3	1, 3
21	CSS	IS	2013	Thailand spec.^d^	*n* = 785/—	16–30; 20.8 ± 2.8	20.8^3^	29.0***	—	—	—	1, 3
21	CSS	IS	2013	Tunisia spec.^d^	*n* = 961/—	16–30; 20.8 ± 2.8	26.3^3^	23.8***	—	—	—	1, 3
21	CSS	IS	2013	Turkey spec.^d^	*n* = 795/—	16–30; 20.8 ± 2.8	18.7^3^	22.5***	—	—	—	1, 3
40	CSS	MS	1985Ŧ	US nat.	*n* = 170,971/52.8	≥18; —	23.9^3^	35.0	—	—	—	—
41	CSS	TS	1985–1988ŦŦ	US nat.	*n* =117,827/50.8	≥18; —	20.0^3^	38.6*	—	—	—	—
42	CSS	IS	1987–1988	US spec.^e^	*n* = 4,647/54.7	≥18; 37.9 ± 0.21	—	19.7	—	—	75.0	1, 3
43	CSS	IS	1988–1994ŦŦŦ	US nat.	*n* = 13,092/51.9	≥20; 45.7 ± 0.83	—	45.9	—	—	—	—
44	CSS	TS	1989ŦŦ	US nat.	*n* = 64,637/56.6	≥18; 45.0 (no SD)	—	33.5*	28.2*	61.7*	82.0	—
41	CSS	TS	1989/1991–1992	US nat.	*n* = 114,025/—	≥18; —	23.3^3^	33.7*	—	—	—	—
45	CSS	IS	1990	US nat.	*n* = 31,347/—	≥25; —	—	31.7*	—	—	86.3	—
46	CSS	TS	1991	US nat.	*n* = 7,805/—	≥18; —	—	19.3	—	—	72.0	—
47	CSS	TS	1991	US reg.	*n* = 2,072/61.5	≥18; —	—	34.4*	—	—	82.4	—
48	CSS	IS	1994–1998	US reg.	*n* = 123/73.2	18–70; —	67.0^3^	48.8	—	—	29.0	—
49	CSS	TS	1996ŦŦ	US nat.	*n* = 107,804/50.4	≥18; —	—	36.4*	33.7*	70.1*	—	—
50	CSS	TS	1996	US reg.	*n* = 3,010/61.0	≥18; —	59.0^3^	38.0	—	—	60.5	—
51	CSS	TS	1996–1997	US nat.	*n* = 1,760/100	≥40; —	52.8/47.2	64.8	—	—	87.3	—
52	CSS	IS	1997–1998	US reg.	*n* = 3,832/62.0	≥18; 43.5 ± 15.6	—	—	—	52.7	66.0	—
53	CSS	IS	1998Ŧ	US nat.	*n* = 30,433/55.5	≥18; —	—	30.9*	—	—	73.9	—
54	CSS	TS	1999	US reg.	*n* = 1,232/—	≥20; —	—	36.1*	34.1*	70.2*	—	—
55	CSS	TS	1999–2002ŦŦŦ	US nat.	*n* = 5,608/48.3	≥20; 51.5 (no SD)	53.6/46.4	51.2	—	—	—	—
56	CSS	TS	2000ŦŦ	US nat.	*n* = 164,187/57.6	≥18; —	36.4/20.7	39.5*	—	—	48.9	—
57	CSS	IS	2000Ŧ	US nat.	*n* = 17,317/52.0	≥18; —	35.0/18.0	30.0	21.0	51.0	83.0	—
58	CSS	IS	2001–2002ŦŦŦ	US nat.	*n* = 4,354/49.3	≥20; —	37.3/30.2	40.9	10.4	51.3	81.0	—
59	CSS	IS	2001–2006ŦŦŦ	US nat.	*n* = 4,021/—	≥20; —	0.0/100	63.0	—	—	—	—
60	CSS	IS	2002	US spec.^a^	*n* = 210/74.0	—; 52.0 (no SD)	8.0/92.0	49.8	—	—	97.7	1, 2, 3, 4
61	CSS	IS	2002–2003	US spec.^d^	*n* = 38,204/65	18–25; 20.3 ± 1.71	21.0/7.0	49.8	23.4	73.2	57.0	1, 3, 4
62	CSS	MS	2003	US nat.	*n* = 3,771/59.4	≥18; —	35.0/32.5	58.0	—	—	69.0	—
63	CSS	TS	2003ŦŦ	US nat.	*n* = 111,456/52.9	≥20; 51.8 ± 14.5	60.1/39.9	55.7	—	—	—	—
64	CSS	IS	2003–2006ŦŦŦ	US nat.	*n* = 4,784/46.6	≥20; 48.1 ± 0.51	100^3^	47.4	—	—	—	—
65	CSS	IS	2003–2008ŦŦŦ	US nat.	*n* = 16,720/51.0	≥18; —	33.5/32.7	37.0	11.4	48.4	—	—
66	CSS	IS	2005	US spec.^e^	*n* = 813/79.0	18–65; —	32.0/35.2	62.7	—	—	56.0	1, 3, 4
67	CSS	IS	2005–2008ŦŦŦ	US nat.	*n* = 5,474/47.2	20–64; —	47.5/52.5	50.2*	—	—	—	—
68	CSS	IS	2007–2010ŦŦŦ	US nat.	*n* = 9,569/51.0	≥20; —	35.0/33.0	43.0	—	—	—	—
69	CSS	TS/MS	2008ŦŦŦŦ	US nat.	*n* = 7,059/50.8	≥18; 46.1 (no SD)	34.4/27.8	53.4*	—	—	31.0	—
70	CSS	IS	2009	US spec.^a^	*n* = 3,949/65.3	≥18; 39.9 ± 0.88	28.8/47.5	60.1**	—	—	71.0	2
71	CSS	MS	2009–2010	US spec.^g^	*n* = 1,510/74.0	≥18; —	26.0/46.0	51.0	—	—	24.0	4
72	PCS	MS	2012ŦŦŦŦ	US nat.	*n* = 3,407/49.8	≥18; —	—	52.0*	24.0*	76.0*	40.0	—
73	CSS	OS	—	US nat.	*n* = 4,023/100	25–45; 35.2 ± 5.9	—	67.2	—	—	—	—
74	CSS	IS	—	US spec.^a^	*n* = 414/66.3	19–79; 55.0 ± 15.4	0.0/100	73.0	—	—	—	1, 2, 3, 4
21	CSS	IS	2013	Venezuela spec.^d^	*n* = 444/—	16–30; 20.8 ± 2.8	20.5^3^	19.2***				1, 3
(**b**)												
75	CSS	IS	2001–2003	Holland reg.	*n* = 1,441/58.8	35–60; 45.4 ± 6.5	62.4/24.8	38.8*	12.9*	51.7*	60.0	1, 3
76	CSS	IS	2002	Norway, reg.	*n* = 629/42.3	30–60; 42.4 (no SD)	49.1/15.5	27.5*	—	—	44.0	—
77	CSS	TS	1990	US reg.	*n* = 1,445/67	≥18; 45.2 ± 18.1	43.8^3^	66.0**	—	—	81.1	—
78	CSS	IS	1992–1993	US reg.	*n* = 1,143/100	24–42; 31.6 ± 3.8	—	9.9*	—	—	—	—
79	CSS	TS	1994	US reg.	*n* = 244/55.7	≥18; —	—	41.9	—	—	91.1	3
80	CSS	IS	1985	US spec.^e^	*n* = 500/100	25–65; 40.6 ± 10.1	39.0	40.0	—	—	—	1, 2, 3, 4
81	CSS	TS	2003	US spec.^h^	*n* = 572/71.2	≥18; 53.9 ± 15.7	—	49.4	—	—	56.8	1, 2, 3, 4
82	CSS	IS	2003	US spec.^d^	*n* = 392/69.0	—; 23.7 ± 5.5	28.6/15.6	38.3	—	—	—	1, 2, 3, 4
83	CSS	IS	2003–2004	US spec.^i^	*n* = 585/49.0	≥18; 45.9 (no SD)	100^3^	58.0	—	—	—	1, 2, 3, 4
84	CSS	IS	2009	US spec.^i^	*n* = 413/100	≥18; 35.6 ± 14.7	25.0/41.0	59.0	—	—	—	1, 2, 3, 4
85	CSS	IS	—	US spec.^i^	*n* = 203/100	20–64; 33.8 (no SD)	65.7^3^	29.1	40.4	69.5	—	1, 2, 3, 4
86	CSS	IS	—	US spec.^i^	*n* = 219/58.0	—; 31.0 (no SD)	55.5^3^	33.3	41.1	74.4	—	1, 2, 3, 4
Sample size weighed mean								39.1	30.3	66.6		
Only (a)								38.6	30.3	66.7		
Only (b)								41.7	19.2	56.3		

1 Body mass index ≥ 25 and <30 kg/m^2^ (≥23 and <25 kg/m^2^ for 21, 39).

2 Body mass index ≥30 kg/m^2^ (≥25 kg/m^2^ for 21, 39) or 120% of ideal body weight.

3 Overweight plus Obesity.

4 Or avoid gaining weight.

(**a**) General population studies.

(**b**) Ethnic‐minority population studies.

CSS, cross‐sectional survey; IS, in‐person/self‐administered survey; MS, mail survey; OS, online survey; PCS, prospective cohort study; TS, telephone survey.

nat., national representative data; reg., regional representative data; spec., specific setting data.

a Patients from Health/Primary Care Centers.

b Individuals from low socioeconomic areas.

c Individuals from high and low socioeconomic areas.

d University students/community.

e Workers/employees.

f Stationary individuals in a shopping center.

g Patients covered by National Healthcare Insurance.

h Members of religious congregations.

i Members of the community at large.

* Estimated percentages based on the data available.

** Prevalence reported only for overweight and obese individuals.

*** Prevalence reported only for underweight and normal weight individuals.

Ŧ Data from the National Health Interview Survey (NHIS).

ŦŦ Data from the Behavioral Risk Factor Surveillance System (BRFSS).

ŦŦŦ Data from the National Health and Nutrition Examination Survey (NHANES).

ŦŦŦŦ Data from the Health Information National Trends Survey (HINTS).

1 Sample was not representative.

2 Participants were not recruited from an appropriate source and/or no random selection was used to recruit them.

3 No sample size calculation.

4 No information on non‐response/refusals and/or no comparison between responders and non‐responders was made (if there was oversampling and data was weighed to reflect country/region population estimates, we assumed that non‐response was taken into account).

Limitations of the studies were determined with The Joanna Briggs Institute Critical Appraisal Checklist for Studies Reporting Prevalence Data 8.

### Methodological appraisal

Table 1 shows the limitations regarding methodological quality of the included studies. In 22 (general population: 14; ethnic minorities: 8) of the 72 included studies, the population was not representative of the country, region or setting where the studies were conducted (Category 1). In 15 studies (general population: 8; ethnic minorities: 7), participants were not randomly selected and/or were not recruited from an appropriate source (Category 2). Sample size calculation (Category 3) was not performed in 25 studies (general population: 16; ethnic minorities: 9). Furthermore, in 23 studies (general population: 16; ethnic minorities: 7) there was no information on response/refusals rate and/or no comparison between responders and non‐responders (Category 4). All studies presented a standard criterion for the measurement of weight control attempts and therefore none presented limitations in this regard (Category 5).

### Prevalence of weight control attempts

Prevalence rates of weight *loss* and weight *maintenance* attempts varied widely across studies, ranging from 9.5% 21 to 73.8% 33 and 10.4% 58 to 42.1% 36, respectively (Table 1). Five studies did not report separate prevalence rates of weight loss and maintenance attempts. The overall prevalence of weight control (i.e. loss plus maintenance) attempts ranged between 37% 22 and 81.5% 36.

#### General population studies

**Figure 2:**
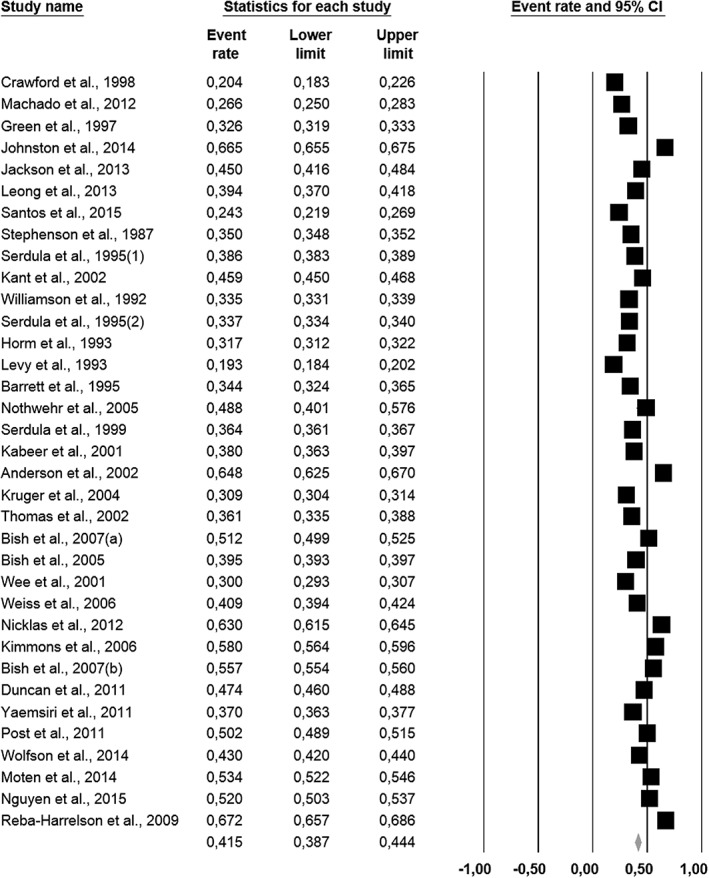
Forest plot for prevalence estimates of weight loss attempts in general populations excluding studies with methodological limitations (*k* = 34).

The overall summary prevalence of weight *loss* and *maintenance* attempts in general populations was 34.6% (95% CI [32.7%, 36.5%]; *Q* = 36,355, *p* < 0.001; *I* ^2^ = 99.8%) and 24.7% (95% CI [23.7%, 31.7%]; *Q* = 5,737, *p* < 0.001; *I* ^2^ = 99.8%), respectively. *Sensitivity analysis* showed that excluding studies with limitations in categories 1 and 3 led to substantial changes in the overall prevalence estimates of weight *loss* attempts in general populations: +7.0% (*k* = 44) and +7.5% (*k* = 42), respectively. Excluding studies with limitations in categories 2 and 4 led to minimal changes: −0.9% (*k* = 50) and −1.7% (*k* = 42), respectively. Figure 2 presents the overall results excluding all studies with limitations in any methodological category. The pooled estimate for the prevalence of weight *loss* attempts in general populations was 41.5% (95% CI [38.7%, 44.4%]; *Q* = 27,947, *p* < 0.001; *I* ^2^ = 99.9%; *k* = 34).

For the overall prevalence estimates of weight *maintenance* attempts in general populations, excluding studies with limitations led to minimal changes (from −1.5% to +0.1%). The combined estimate excluding studies with limitations in any methodological category was 23.2% (95% CI [18.8%, 28.3%]; *Q* = 4,838, *p* < 0.001; *I* ^2^ = 99.8%; *k* = 10).

Regarding *publication bias*, visual inspections of the funnel plots did not show the presence of asymmetry, which was confirmed with Egger's test (all *p* > 0.05), either for the prevalence of weight *loss* or *maintenance* attempts in general populations.

Table 2 presents the results of the subgroup analyses. A clear significant increase in the prevalence of weight loss attempts across decades is observed (from 18.6% to 47.7%, *Q* = 138.454, *p* < 0.001), until the decade of 2000–2009. Between 2010 and 2015 the combined prevalence was 24.1%. High heterogeneity and large proportion of dispersion in the prevalence rates was observed within subgroups (*I* ^2^ varied between 98.8% and 99.8%). *Sensitivity analysis* showed that excluding studies with any methodological limitation led to important changes in the overall prevalence estimates of weight *loss* attempts in the decade of 2010–2015 (increased to 39.7%). For the other decades, changes were small.

**Table 2:** Subgroup analysis assessing the effect of pre‐selected moderators on the prevalence of weight loss attempts in general populations

Moderators	*N* analyses	Prevalence (%) [95% CI]	*Q* 1	*p* 1	*I* ^2^ (%)
Decade of survey			138.454	<0.001	
1970–1979	1	18.6 [17.7, 19.5]			0.0
	**0**	—			—
1980–1989	4	35.8 [35.6, 35.9]			99.7
	**3**	**35**.**9** [**35**.**8**, **36**.**1**]			**99**.**7**
1990–1999	18	34.4 [31.0, 37.9]			99.6
	**13**	**35**.**5** [**31**.**7**, **39**.**4**]			**99**.**6**
2000–2009	24	47.7 [44.4, 50.9]			99.8
	**14**	**48**.**2** [**44**.**2**, **52**.**2**]			**99**.**9**
2010–2015	29	24.1 [21.9, 26.4]			98.8
	**4**	**39**.**7** [**38**.**6**, **40**.**9**]			**99**.**4**
Geographic region			108.335	<0.001	
Africa	6	16.6 [12.7, 21.4]			79.5
	**0**	—			—
East Asia and Pacific	15	33.1 [28.9, 37.6]			98.3
	**2**	**31**.**7** [**30**.**0**, **33**.**4**]			**99**.**2**
Europe and Central Asia	10	31.5 [26.6, 36.8]			99.8
	**3**	**61**.**3** [**60**.**4**, **62**.**3**]			**99**.**7**
Latin America and the Caribbean	6	20.6 [15.9, 26.1]			98.5
	**1**	**26**.**6** [**25**.**0**, **28**.**3**]			**0**.**0**
Middle East and North Africa	3	26.8 [25.4, 28.2]			93.1
	**0**	—			—
North America	37	44.0 [41.0, 47.1]			99.9
	**29**	**42**.**6** [**39**.**6**, **45**.**7**]			**99**.**9**
South Asia	3	25.6 [23.8, 27.5]			96.1
	**0**	—			—
*Meta‐regressions*	*N analyses*	*Slope*, *SE* [*95*% *CI*]	*Z*	*p*	
% Overweight and obesity	70	0.018, 0.001 [0.016, 0.020]	14.869	<0.001	
	**26**	**0**.**015**, **0**.**002** [**0**.**011**, **0**.**018**]	**8**.**727**	<**0**.**001**	
% Women	58	0.011, 0.003 [0.004, 0.017]	3.260	0.001	
	**30**	**0**.**012**, **0**.**004** [**0**.**003**, **0**.**020**]	**2**.**625**	**0**.**009**	
Mean age	48	0.038, 0.007 [0.024, 0.051]	5.490	<0.001	
	**11**	**0**.**001**, **0**.**034** [−**0**.**067**, **0**.**068**]	**0**.**020**	**0**.**984**	

1 Cochran's *Q* statistic and *p*‐values correspond to subgroup differences in effects. Results from sensitivity analyses are represented in bold.

There were significant differences in the prevalence of weight loss attempts between geographic regions (*Q* = 108.335, *p* < 0.001), in which the highest overall prevalence was found in North America (44%, 95% CI [41%, 47.1%]) and the lowest in Africa (16.6%, 95% CI [12.7%, 21.4%]) (Table 2). All subgroups presented significant heterogeneity and large proportion of dispersion in the prevalence rates (*I* ^2^ varied between 93.1% and 99.9%). *Sensitivity analysis* showed that the prevalence of weight loss attempts in Europe and Central Asia and in Latin America and the Caribbean, excluding studies with any methodological limitation, was much higher: from 31.5% to 61.3% and from 20.6% to 26.6%, respectively. Slight changes were observed in East Asia and Pacific and in North America (−1.4%).

Combined prevalence of weight loss attempts increased significantly with the prevalence of overweight and obesity (*b* = 0.018; *p* < 0.001), with the percentage of women in the samples (*b* = 0.011; *p* = 0.001) and with mean age (*b* = 0.038; *p* < 0.001). *Sensitivity analysis* showed that when excluding all the studies with methodological limitations, only the association between weight loss attempts and mean age became non significant.

#### Ethnic‐minority population studies

Overall results of the meta‐analysis for the prevalence of weight *loss* attempts in ethnic minorities showed a pooled estimate of 39.6% (95% CI [29.7%, 50.4%]; *Q* = 867.199, *p* < 0.001; *I* ^2^ = 98.7%). Combined prevalence of weight *maintenance* attempts was 21.1% (95% CI [19.2%, 23.2%]; *Q* = 147,583, *p* < 0.001; *I* ^2^ = 98.6%). *Sensitivity analysis* showed that excluding all studies with methodological limitations led to an increase in the overall prevalence of weight *loss* attempts of 4.5% (44.1%; *k* = 3). All of the studies reporting *maintenance* attempts presented methodological limitations.

Subgroup analyses by decade of survey showed a prevalence of weight loss attempts of 40% between 1980 and 1989, 48.5% between 1990 and 1999 and 44.9% between 2000 and 2009 (Table 3). Subgroup analyses by geographic region revealed a prevalence of weight loss attempts of 35.6% in Europe and Central Asia and 41% in North America (Table 3). For both analyses, there were no significant differences between groups (*Q* = 0.415, *p* = 0.813 and *Q* = 0.305, *p* = 0.581, respectively). Meta‐regressions by mean age and percentage of women in the samples were also not significant (*b* = 0.042; *p* = 0.118 and *b* = −0.009; *p* = 0.440, respectively). Because only three studies did not present methodological limitations, we did not conduct *sensitivity analyses* for this set of moderation analyses.

**Table 3:** Subgroup analysis assessing the effect of pre‐selected moderators on the prevalence of weight loss attempts in ethnic‐minority populations

Moderators	*N* analyses	Prevalence (%) [95% CI]	*Q* 1	*p* 1	*I* ^2^ (%)
Decade of survey			0.415	0.813	
1980–1989	1	40.0 [35.8, 44.4]			99.7
1990–1999	3	48.5 [46.3, 50.7]			99.6
2000–2009	6	44.9 [28.1, 62.8]			99.8
Geographic region			0.305	0.581	
Europe and Central Asia	2	35.6 [33.5, 37.7]			95.9
North America	10	41.0 [29.5, 53.5]			98.8
*Meta‐regressions*	*N analyses*	*Slope*, *SE* [*95*% *CI*]	*Z*	*p*	
% Women	12	−0.009, 0.011 [−0.030, 0.013]	−0.771	0.440	
Mean age	11	0.042, 0.027 [−0.011, 0.095]	1.561	0.118	

1 Cochran's *Q* statistic and *p*‐values correspond to subgroup differences in effects.

### Personal weight control strategies

Twenty‐seven studies (25 general population studies and 2 ethnic‐minority population studies) reported strategies used by those trying to control their weight (Table 4). Thirty‐seven strategies were identified across studies, which were grouped in 10 domains of the OxFAB Taxonomy. Doing or increasing physical activity – the only strategy that fell in the *energy compensation* domain – was the most frequently assessed strategy (*k* = 27 for trying to *lose* and *k* = 7 for trying to *maintain* weight), and results show that this strategy was used by the majority of participants across studies. Dieting – within the *restraint* domain – was the second most assessed strategy for trying to *lose* weight (*k* = 20) and was even more frequently reported: more than two‐thirds of participants attempted to lose weight using this strategy. All other strategies were assessed by 1 to 14 studies. The domain that combined more strategies was the *regulation – restrictions* (*k* ranged between 1 and 9): from 12% to 66% of participants and from 2% to 64% of participants reported avoiding or restricting specific foods or behaviours for trying to *lose* and *maintain* weight, respectively. *Dietary choices* (*k* = 1–4) and *weight management aids* (*k* = 1–14) were the other domains were more strategies fell on: from 39% to 85% of participants reported choosing specific dietary behaviours and from 1% to 25% of participants reported using some aid to try to *lose* weight; from 36% to 87% of participants reported choosing specific dietary behaviours and from 1% to 6% of participants reported using some aid to try to *maintain* weight.

**Table 4:** Personal weight control strategies

	Weight loss attempts				Weight maintenance attempts			
Domains Strategies	Number of studies	*n*	Prevalence (%)*	References	Number of studies	*n*	Prevalence (%)*	References
**Dietary choices**								
Eat/drink low‐calorie foods/beverages^1^	4	4,285	39.2	46, 58, 59, 86	1	396	35.9	58
Drink water	3	4,445	38.7	38, 58, 59	2	609	40.2	38, 58
Eat more/regularly fruits and vegetables^2^	3	374	85.3	38, 52, 86	2	213	86.9	38, 52
Eat breakfast	1	267	63.7	38	1	213	69.0	38
Eat soup	1	267	70.8	38	1	213	73.2	38
**Energy compensation** **								
Increased/regular PA/Exercise^2^, ^3^, ^4^, ^5^	27	122,314	65.2	15, 16, 19, 20, 23, 24, 32, 34, 35, 38, 40, 46, 47, 49, 50, 52, 53, 55, 56, 58, 59, 63, 65, 70, 74, 77, 86	7	36,000	50.2	15, 16, 38, 49, 52, 58, 65
**Information seeking**								
Select foods consciously	3	692	82.2	15, 16, 38	3	679	83.7	15, 16, 38
Seek information on food/nutrition/PA^5^	2	267	57.7	35, 38	1	213	57.7	38
**Regulation**: **Restrictions**								
Skip meals^2^, ^4^, ^6^	9	15,135	13.8	15, 16, 24, 46, 52, 53, 58, 59, 86	4	396	8.90	15, 16, 52, 58
Eat less fat/fatty foods^2^	6	13,895	51.0	16, 52, 53, 58, 59, 86	3	615	57.9	16, 52, 58
Eat less sugar/sugary foods^2^, ^3^	4	298	66.1	16, 20, 52, 86	2	219	63.5	16, 52
Follow a special/fad diet	4	5,876	11.9	38, 46, 58, 59	2	609	1.64	38, 58
Drink less alcoholic beverages	2	425	44.9	15, 16	2	466	42.5	15, 16
Eat less fried/junk foods^2^	1	—	—	52	1	—	—	52
Eat less high‐carbohydrate foods	1	107	60.7	86	0			
Eat less meat	1	107	49.5	86	0			
Limit snacking	1	107	59.8	86	0			
**Regulation**: **Rule‐setting**								
Eat more frequently (small meals)	2	1,698	20.7	38, 46	1	213	69.0	38
Eat slowly	1	267	47.6	38	1	213	50.7	38
Choose small portions	1	267	67.6	38	1	213	67.6	38
**Restraint**								
Dieting^2^, ^3^, ^4^, ^5^	20	117,337	68.9	19, 20, 23, 24, 32, 34, 35, 40, 46, 47, 49, 50, 53, 55, 56, 63, 65, 70, 74, 77	2	34,925	65.9	49, 65
Reduce amount of food eaten^2^, ^3^	7	4,710	66.9	15, 16, 20, 52, 58, 59, 86	4	862	63.3	15, 16, 52, 58
**Self‐monitoring**								
Count calories^3^	5	2,123	20.4	15, 16, 20, 38, 46	3	679	6.92	15, 16, 38
Record dietary intake and PA	2	1,698	12.4	38, 46	1	213	7.04	38
Self‐weighing	1	1,431	70.7	46	0			
**Support**: **Professional**								
Attend a weight control programme or group^4^	9	16,585	6.28	15, 19, 24, 34, 38, 46, 53, 58, 59	3	856	1.87	15, 38, 58
Receive advice from a healthcare professional^5^	3	934	13.2	19, 35, 38	1	213	16.4	38
**Weight management aids**								
Use weight loss pills or supplements^2^, ^4^	14	19,008	10.2	16, 19, 23, 24, 32, 34, 35, 38, 46, 52, 53, 58, 59, 86	4	828	1.21	16, 38, 52, 58
Use laxatives or diuretics^2^, ^7^	10	13,067	2.92	15, 16, 34, 35, 38, 46, 52, 53, 58, 86	5	679	6.19	15, 16, 38, 52, 58
Eat diet foods or products^1^, ^5^	7	5,528	15.9	15, 23, 34, 35, 46, 58, 59	2	643	3.42	15, 58
Use meal replacements (food/drinks)	5	6,189	9.73	16, 34, 46, 58, 59	2	615	1.30	16, 58
Vitamins	3	2,547	25.2	23, 34, 46	0			
Traditional medicine^5^	2	389	10.0	34, 35	0			
Devices	1	1,431	0.98	46	0			
Surgery	1	1,431	0.56	46	0			
**Extreme strategies**								
Fasting or vomiting^2^, ^6^, ^7^	10	12,856	4.74	15, 16, 32, 35, 38, 46, 52, 53, 58, 86	5	213	0.90	15, 16, 38, 52, 58
Smoking	3	532	6.95	15, 16, 86	2	466	5.15	15, 16

* Prevalence indicates the number of respondents out of *n* study sample that reported using strategies for trying to lose or maintain weight.

1 Study 46 was not accounted for sample size or prevalence rate in this strategy because of assessment differences (several low‐calorie foods were assessed separately).

2 Studies 52, 65 were not accounted for sample size or prevalence rates because they did not report separate values for trying to lose and maintain weight.

3 Studies 20, 40, 70, 77 were not accounted for sample size or prevalence rates because of assessment differences (only the key method was assessed).

4 Studies 24, 55, 63, 74 were not accounted for sample size or prevalence rates because they did not have sufficient data available.

5 Study 35 was not accounted for sample size or prevalence rates in this strategy because of assessment differences (only the key method was assessed).

6 Studies 15, 16 were not accounted for sample size or prevalence rates in this strategy because of assessment differences (fasting was assessed together with skipping meals).

7 Study 58 was not accounted for sample size or prevalence rate in this strategy because of assessment differences (vomiting was assessed together with the use of laxatives).

** Exercise/physical activity was considered in the Energy Compensation domain because this strategy is commonly used to compensate energy intake as a way to control weight.

### Weight control motives

Of the 72 included studies, only seven from general populations reported motives for trying to lose and/or maintain weight (Table 5). *To improve appearance* and *to improve health and prevent future diseases* were the most frequently assessed motives for trying to *lose* weight (*k* = 5), although *to improve wellbeing* was the most frequently reported motive (95%), followed by *to improve fitness condition or staying fit* (85%) and *to improve self‐esteem* (74%). The most frequently reported motive for trying to *maintain* weight was *to improve health and prevent future diseases* (98%), followed by *to improve wellbeing* (91%), *to improve fitness condition or staying fit* (87%), *to improve appearance* (80%) and *to improve self‐esteem* (71%). All other motives (e.g. *to please or by insistence of spouse*/*partner*, *because of health professional advice*) were listed by less than 50% of participants.

**Table 5:** Weight control motives

	Weight loss attempts				Weight maintenance attempts			
Motives	Number of studies	*n*	Prevalence (%)*	References	Number of studies	*n*	Prevalence (%)*	References
Improve appearance	5	1,104	71.4	15, 24, 32, 35, 38	2	460	79.8	15, 38
Improve health/prevent diseases	5	1,104	35.3	15, 24, 32, 35, 38	2	460	97.6	15, 38
Improve wellbeing	3	501	95.0	15, 22, 38	3	460	90.8	15, 22, 38
Improve fitness condition/stay fit	3	501	84.6	15, 32, 38	2	460	86.9	15, 38
Improve self‐esteem	3	501	73.9	15, 32, 38	2	460	71.0	15, 38
Health professional advice	3	501	40.0	15, 22, 38	3	460	38.6	15, 22, 38
Please/insistence of spouse or partner	2	234	46.2	15, 22	2	247	32.0	15, 22
Improve social life/avoid discrimination	2	267	37.8	22, 38	2	213	44.6	22, 38
Improve professional life/fulfil specific professional requirements	2	267	32.2	22, 38	2	213	42.7	22, 38
Please/insistence of family	2	234	27.8	15, 22	2	247	24.9	15, 22
Decrease disease risk (e.g. heart attack)	1	—	—	24	0	—	—	
Special event/season (e.g. holiday, summer)	1	—	—	22	1	—	—	22

* Prevalence indicates the number of respondents out of *n* study sample that reported motives for trying to lose or maintain weight. Studies 22, 24, 35 were not accounted for sample size or prevalence rate because they did not have sufficient data available. Data from study 46 was not included because of methodological differences (only the most important motive was reported).

## Discussion

This comprehensive systematic review and meta‐analysis sought to estimate the prevalence of weight control attempts among adults worldwide, and identify potential correlates, personal strategies used and the underlying motivations. Seventy‐two studies with more than a million participants were included, showing that weight is a matter of concern to a significant portion of the population. Results from high quality studies showed that about 42% of adults from general populations and 44% of adults from ethnic‐minority populations reported trying to *lose* weight, and about 23% of adults from general populations reported trying to *maintain* weight at some point in time. Significant differences were found between decades and geographic regions: higher prevalence rates of weight loss attempts among adults from general populations occurred in the decade of 2000–2009 and in Europe and Central Asia. In the last five years (2010–2015), about 40% of adults from general populations reported trying to lose weight. As expected, higher prevalence of weight loss attempts among adults was observed in overweight and obese persons and in women. Across populations, 37 different personal strategies were reported for managing weight, standing out physical activity participation and dieting, which were classified, respectively, within the energy compensation and restraint domains of the OxFAB Taxonomy. Finally, 12 different motives for trying to manage weight were cited, the most common being increasing wellbeing and achieving long‐term health.

To our knowledge, this is the first systematic review and meta‐analysis presenting comprehensive estimates of the prevalence of weight *loss* and *maintenance* attempts and describing the related factors among adults across the globe. This is of considerable relevance because accurate information in this area should assist in the evaluation of changes and trends worldwide, in setting priorities for public health initiatives and in planning management of weight control services.

### Prevalence of weight control attempts

The overall summary of prevalence results in general populations mirrors the overweight and obesity trends worldwide: prevalence rates have increased in the last decades and are higher in Europe/Central Asia and in the US 87. The prevalence of weight loss attempts appears to have peaked in the beginning of the 2000s. Factors that explain the growing prevalence of weight loss attempts in the start of this millennium may include changes in social norms regarding obesity, an increase in the number of products and services targeting weight management or greater importance attributed by the population to weight or body shape and health. The significant differences observed between geographic regions may also be linked to the cultural context of each region, as well as to the physical environment and socioeconomic condition, as these factors may influence the development of health‐promoting behaviours. For example, individuals from higher socioeconomic groups and with higher levels of education are more likely to try to control their weight 88, 89, perhaps because they are exposed to social advantages such as access to weight loss services, higher affordability of healthy choices and knowledge, which collectively facilitate the adoption of energy‐balance related behaviours 90, 91. Our findings also highlight the role of gender on weight‐related aspects, with more women attempting to lose weight than men. One possible explanation for this is that social norms and cultural pressures to be thin especially affect women 92, or that women with normal weight often perceive themselves as being overweight 65 and consequently engage in more efforts to become or remain thin.

Although the prevalence of overweight and obesity is particularly high in some ethnic‐minority groups 93, the overall summary prevalence of weight loss attempts in this population was only slightly different than that observed in general populations (+2.6%), apparently presenting its peak also in the beginning of the 2000s. One possible justification is a difference in attitudes and cultural norms regarding weight: for example, previous studies have shown that non‐Hispanic black and Hispanic women are more satisfied with their body size than Caucasian women, and individuals who are satisfied with their body size are less likely to try to lose weight 94. However, it should be noted that the limited number of studies without methodological limitations (*k* = 3, *n* = 3,217) reduces the confidence in the results, compared with the analyses with general populations (*k* = 34, *n* = 1,062,133). Also, the limited number of studies with ethnic‐minority populations limits the conclusions that can be drawn from the moderator analyses because we could not test whether the prevalence of weight loss attempts varied with the prevalence of overweight and obesity, and the non‐significant effects found may be due to low statistical power 10.

### Personal weight control strategies

The most consistently assessed strategies, which were used by the majority of individuals trying to lose and maintain weight, were related with increasing energy expenditure and reducing energy intake, in line with evidence‐based guidelines for weight management 1. Other strategies related with improving the quality of the diet – by increasing the consumption of healthy foods (e.g. eating more fruits and vegetables) or restricting the consumption of unhealthy foods (e.g. eating less sugary foods) – were moderately assessed across studies, and also frequently reported, although they may or may not have an impact on body weight (e.g. 95). Importantly, several weight management aids – weight loss pills or supplements, laxatives or diuretics, diet products and meal replacements – and also more extreme strategies – fasting or vomiting – were reported by a low percentage of individuals, mostly for trying to lose weight. This is encouraging in suggesting that in the adult population worldwide, weight control appears to be more associated with health‐promoting rather than potentially harmful strategies. With the exception of a few of these strategies (e.g. some weight loss pills 96), there is no scientific evidence suggesting their effectiveness (e.g. 59, 97, 98), and they may be associated with weight cycling and regain over time 99, and with eating disorders 100.

### Weight control motives

Based on the present findings, the public health message on managing weight for long‐term health, preventing disease, general wellbeing or improving fitness seem to have been endorsed across populations. However, reasons such as enhancing appearance, conforming to external request/demands (spouse or doctor), and avoiding discrimination were also relatively common. Considering the positive role that more internal motives (e.g. health and wellbeing) appear to have on long‐term weight control and related behaviours 101, 102, the large endorsement of relatively external motives (e.g. social pressure, even protecting one's self‐esteem from prejudice) may be a cause for concern. Research has now clearly shown that having a more positive body image, not feeling pressured or discriminated against, and losing weight mostly for autonomous (i.e. more internal) reasons pay off in increased adherence to weight‐healthy behaviours and higher success rates 103, 104, 105.

### Strengths and limitations

The present review has a number of strengths but also some limitations. First, while it includes a large number of epidemiological studies, which make the findings robust, they are not all nationally representative and response rates varied considerably among studies, leading to inexact overall prevalence rates. Although nationally representative samples are preferable, not including the regional representative and the non‐representative samples would limit our results in terms of time points and geographical regions for which nationally representative data is not available. We performed sensitivity analyses, repeating the analyses without the studies with methodological limitations, in an attempt to partially overcome this limitation.

Second, although surveys have several advantages – they are the standard way of gathering prevalence data, are relatively cheap to administer, information is uniform across the years and privacy can be maintained – they also have disadvantages. These include being subject to social desirability and selection bias towards more motivated individuals; they are also sensitive to the target groups' literacy level 106. Also, surveys were administered in different ways across studies – electronically, via mail, telephone or in person – which could impact final results. Third, although important potential moderators of weight control attempts were tested – percentage of women in the samples, percentage of overweight individuals in the samples, age, geographic region and decade of survey – other variables with potential moderator effect were not assessed, such as socioeconomic status or education level. The main reason for not including these variables was the different methodologies to assess these constructs used across studies, which makes it difficult to standardize results. Fourth, a great variety of personal strategies for attempting to control weight were assessed in a relatively small number of studies, which makes it difficult to determine the exact prevalence of usage of each method. This makes results for weight loss strategies less robust that what is desirable, which should be seen as an important limitation. A systematic standardized approach in this regard must be taken in future studies (for example, using validated instruments like the OxFAB taxonomy 13, which we have used to synthesize results). Finally, weight control motives, which are particularly susceptible to social desirability/undesirability, were assessed in only a small number of studies and without a standardized instrument, probably leading to over‐/underestimated results.

### Conclusions and implications

In summary, this study indicates that in the general population about four in 10 adults have tried to lose weight at some point in time and also in the last five years. Key strategies and motives associated with weight control were identified, presenting a clearer picture of weight management from the perspective of those actively seeking it. Although the majority of individuals used strategies in line with recommendations (social desirability notwithstanding), we could not estimate the psychological, economical or social impacts that these attempts may involve. Losing weight often involves substantial investments of time, energy and expectations, sometimes lasting years or decades, something which cannot be ignored. Finally, there seems to be a mismatch between prevalent motives to lose weight and those which research indicates as more conducive to long‐term success.

We believe that this detailed picture of weight control attempts among adults worldwide provides valuable information for healthcare professionals and policy makers towards better planning and resource allocation directly targeting obesity prevention and treatment. From a societal perspective, and considering the high demand for weight management solutions, it is imperative to rigorously evaluate the quality of community, public, and especially commercial weight management services and products, which are increasingly available, and to respond to this demand by funding research for, and promoting evidence‐based and safe services and products targeting long‐term weight control. Additionally, in order to better capture prevalence shifts, surveillance systems should be scheduled, with internationally widespread screening instruments developed and validated for that purpose that will guarantee accuracy and comparability of results.
